# Caprylic Acid Restores Branched-Chain Amino Acid Metabolism in a Mouse Cachexia Model

**DOI:** 10.3390/cimb47050325

**Published:** 2025-05-01

**Authors:** Isao Kawahara, Rina Fujiwara-Tani, Takuya Mori, Shota Nukaga, Ryoichi Nishida, Yoshihiro Miyagawa, Kei Goto, Hitoshi Ohmori, Kiyomu Fujii, Yi Luo, Takamitsu Sasaki, Chie Nakashima, Ruiko Ogata, Hiroki Kuniyasu

**Affiliations:** 1 Department of Molecular Pathology, Nara Medical University School of Medicine, Kashihara 634-8521, Japan; isao_kawahara@a011.broada.jp (I.K.); pt_mori_t@yahoo.co.jp (T.M.); shota.nukaga@gmail.com (S.N.); g.m__r1@outlook.jp (R.N.); y.miya1103@gmail.com (Y.M.); ilgfgtk@gmail.com (K.G.); brahmus73@hotmail.com (H.O.); toto1999-dreamtheater2006-sms@nifty.com (K.F.); lynantong@hotmail.com (Y.L.); takamitu@fc4.so-net.ne.jp (T.S.); c-nakashima@naramed-u.ac.jp (C.N.); pkuma.og824@gmail.com (R.O.); 2 Division of Rehabilitation, Hanna Central Hospital, Ikoma 630-0243, Japan; 3 Department of Medical Ethics and Genetics, Kyoto University, Kyoto 606-8501, Japan

**Keywords:** cancer sarcopenia, branched-chain amino acids, branched-chain α-ketoacid dehydrogenase, branched-chain α-ketoacid dehydrogenase, caprylic acid

## Abstract

Cancer-associated sarcopenia is closely linked to the prognosis of cancer patients, making its management a critical aspect of cancer treatment. Branched-chain amino acids (BCAAs) are known to promote skeletal muscle growth in healthy individuals; however, their efficacy in cancer patients remains controversial. In this study, we investigated the effects of BCAAs on cancer-associated sarcopenia to identify the underlying mechanisms that may suppress their effectiveness. In both a mouse cachexia model and an in vitro cachexia model, BCAAs did not significantly reduce oxidative stress, improve oxidative phosphorylation, suppress cytokine production, or enhance muscle mass and maturation, as observed in non-cancer-bearing models. Furthermore, treatment with 5-fluorouracil exacerbated sarcopenia in the mouse cachexia model, independent of tumor weight reduction, and this deterioration was not ameliorated by a BCAA-supplemented diet. The ineffectiveness of BCAAs was attributed to impaired BCAA catabolism, characterized by the decreased expression of branched-chain α-ketoacid dehydrogenase (BCKD) and increased levels of its inactive phosphorylated form, which were driven by elevated expression of BCKD kinase. These metabolic alterations were induced by high-mobility group box-1 (HMGB1). Notably, caprylic acid reversed these impairments in BCAA metabolism, thereby restoring BCAA efficacy. Our findings suggest that enhancing BCAA metabolism may improve their therapeutic potential in the treatment of cancer-associated sarcopenia.

## 1. Introduction

Sarcopenia is a progressive muscle disease characterized by muscle failure resulting from cumulative deleterious changes over a lifetime [1]. Cachexia is a condition associated with diseases such as cancer, organ failure, and infections, and is characterized by generalized wasting, including, but not limited to, muscle loss, which is termed sarcopenia [2]. Cancer cachexia affects approximately 80% of patients with advanced cancer and 40% of patients across all cancer stages [3,4,5], with elderly cancer patients being particularly vulnerable [6]. Cancer cachexia is a complex metabolic syndrome involving multiple factors, among which skeletal muscle atrophy is a key pathological feature [7,8,9]. Moreover, chemotherapy and certain targeted therapies have been recognized as contributing factors to sarcopenia, leading to reduced physical function and a diminished quality of life [10]. Notably, in cancer patients, the skeletal muscle mass is closely linked to treatment tolerance [11] and is strongly correlated with the overall survival [12].

Key factors implicated in cancer-associated sarcopenia include cancer-related inflammatory cytokines [13,14], increased oxidative stress [15], enhanced catabolism [16], and anorexia [11]. Studies utilizing a mouse cachexia model have demonstrated that elevated oxidative stress and impaired mitochondrial energy metabolism play crucial roles in the progression of cachexia [17]. Additionally, high-mobility group box-1 (HMGB1), an inflammatory cytokine, has been implicated in promoting oxidative stress, inflammatory responses, autophagy, and skeletal muscle aging [13,14,17].

Amino acids play a crucial role in various nutritional interventions aimed at counteracting cancer-related sarcopenia [18]. In response to the increased oxidative stress associated with cancer, the combined use of the antioxidant amino acids cystine and theanine has been shown to effectively reduce sarcopenia [19]. Conversely, branched-chain amino acids (BCAAs), particularly leucine (Leu), are metabolically activated by peroxisome proliferator-activated receptor gamma coactivator 1-α (PGC-1α), enhancing energy metabolism and stimulating the mechanistic target of rapamycin complex 1 (mTORC1) to promote protein synthesis [20]. Among the BCAAs, Leu plays a particularly significant role, as its blood concentration is directly related to muscle protein synthesis [21]. In addition to its role in muscle maintenance, the BCAA levels are also linked to appetite regulation. A reduction in BCAA levels is associated with increased serotonin synthesis, which can lead to anorexia [22]. Thus, BCAA supplementation has been shown to improve appetite, making it beneficial for aging-related sarcopenia and overall health maintenance [23,24].

Given its strong anti-anorectic and anti-cachectic effects, BCAA is also considered a promising intervention for cancer-associated sarcopenia [25]. However, despite the growing interest in the role of amino acids in skeletal muscle preservation, the specific mechanisms and clinical effectiveness of BCAA in cancer cachexia remain insufficiently explored. In this study, we aim to investigate the effects of BCAA on cancer-related sarcopenia using a mouse model of cancer cachexia and an in vitro cachexia model that we have previously developed [26,27].

## 2. Materials and Methods

### 2.1. Cell Culture

CT26 murine colon carcinoma cells were generously provided by Professor I.J. Fidler (MD Anderson Cancer Center, Houston, TX, USA). C2C12 myoblasts derived from mouse skeletal muscle were obtained from Dainihon Pharmacy Co. (Tokyo, Japan). Both cell lines were maintained in Dulbecco’s Modified Eagle’s Medium (DMEM; Wako Pure Chemical Industries, Osaka, Japan) supplemented with 10% fetal bovine serum (Sigma-Aldrich, St. Louis, MO, USA). C2C12 cells were treated with BCAAs (Leu, Ile, and Val, 200 μM each, Wako), HMGB1 (40 μg/mL, BioLegend Inc., San Diego, CA, USA) or caprylic acid (C8) (50 μg/mL, Wako) [28] for 48 h.

### 2.2. In Vitro Cachexia Model

To model cancer cachexia in vitro [26], the culture medium was supplemented with 20% (*v*/*v*) ascitic fluid collected from BALB/c mice previously inoculated with CT26 cells. For the control condition, 20% (*v*/*v*) CT26 culture supernatant was added to the medium. A comparison of the compositional differences among standard DMEM, ascites-supplemented medium, and culture supernatant-supplemented medium is provided in Table 1. C2C12 cells were treated with ascites-supplemented medium or culture supernatant-supplemented medium for 48 h.

### 2.3. Animals

Five-week-old male BALB/c mice were acquired from SLC Japan (Shizuoka, Japan). Mice were housed in a pathogen-free environment with a 12 h light/dark cycle at 22 °C and controlled humidity. All animal procedures adhered to the institutional guidelines approved by the Animal Ethics Committee of Nara Medical University (Approval No. 12777, 20 April 2020) and were consistent with the Japanese Ministry of Health, Labor and Welfare regulations. Mice were allowed to acclimate for one week prior to experimentation.

The cachexia model was established via intraperitoneal injection of CT26 cells, as previously described [27]. Each experimental group contained five mice. For chemotherapy treatment, 5-fluorouracil (5FU; Wako) was administered subcutaneously at a dose of 30 mg/kg once weekly. The quadriceps femoris muscle (QFM) was isolated post-euthanasia. Tumor burden was evaluated by gross dissection of peritoneal tumor masses from the abdominal wall, mesentery, diaphragm, and intestines.

### 2.4. Dietary Interventions

Mice were fed either a control diet (CE-2; CLEA Japan, Tokyo, Japan), a branched-chain amino acid (BCAA)-supplemented diet, or a diet enriched with both BCAAs and 2% (*w*/*w*) C8 (Wako). Diet formulations are detailed in Table 2. Food and caloric intake per mouse were calculated from the cumulative daily intake of each group (n = 5).

### 2.5. Protein Extraction

Proteins were extracted from C2C12 cells and mouse QFM as previously reported [28]. Whole-cell lysates were prepared in radioimmunoprecipitation assay (RIPA) buffer containing 0.1% SDS (Thermo Fisher Scientific, Tokyo, Japan). Total protein concentrations were measured using the Rapid Protein Assay Kit (Wako).

### 2.6. Western Blotting

Equal amounts of protein were resolved on 10% SDS-PAGE gels and transferred onto nitrocellulose membranes. Membranes were incubated with specific primary antibodies (Table 3), followed by HRP-conjugated secondary antibodies (P0217; Dako, Glostrup, Denmark). Protein bands were visualized using the Fusion Solo imaging system (M&S Instruments, Osaka, Japan).

### 2.7. Enzyme-Linked Immunosorbent Assay (ELISA) and Fluorometric Analysis

The levels of MYL1, HMGB1, TNF-α, GSH/GSSG, 4-hydroxynonenal (4HNE), BCAAs, and acetyl-CoA were quantified using commercial ELISA kits according to the manufacturers’ protocols (Table 3), using whole-cell lysates.

### 2.8. Mitochondrial Stress Test (Seahorse Assay)

Mitochondrial and glycolytic functions were assessed using the Seahorse XFe24 Extracellular Flux Analyzer (Agilent Technologies, Chicopee, MA, USA), as described previously [26]. Oxygen consumption rate (OCR) and extracellular acidification rate (ECAR) were measured in C2C12 cells (1 × 10^4^ cells/well) using the Seahorse XF24 FluxPak (SeahorseBioscience, North Billerica, MA, USA).

### 2.9. Glycolytic Stress Test

Glycolytic activity was analyzed using a modified glycolytic stress test with the Seahorse XFe24 platform. Prior to measurement, C2C12 cells were cultured in 6-well plates with either ascitic fluid or CT26-conditioned medium. For the assay, cells were seeded at 1 × 10^4^ cells/well in XF Base Medium supplemented with 200 mM L-glutamine and 5 mM HEPES. Sensor cartridges were hydrated in XF Calibrant 24 h before use. The following compounds were sequentially injected: glucose (10 mM), oligomycin (1 µM), rotenone and antimycin A (1 µM each), and 2-deoxyglucose (50 mM). The combined rotenone/antimycin injection enabled evaluation of mitochondrial contributions to ECAR through glycolytic intermediates entering the TCA cycle.

### 2.10. Reverse Transcription–Polymerase Chain Reaction (RT-PCR)

Total RNA (0.5 µg) from cultured cells was isolated using the RNeasy Mini Kit (Qiagen, Germantown, MD, USA). RT-PCR was performed using primer pairs listed in Table 3 (synthesized by Sigma Genosys, St. Louis, MO, USA) to evaluate gene expression. PCR products were resolved on 2% agarose gels and visualized by ethidium bromide staining. ACTB mRNA served as an internal reference.

### 2.11. Statistical Analysis

Statistical analyses were conducted using one-way ANOVA with Bonferroni’s post hoc correction in GraphPad InStat software (v3.1; GraphPad Software Inc., La Jolla, CA, USA). Results are presented as mean ± SD from at least three independent experiments. A *p*-value < 0.05 was considered statistically significant. Bonferroni-adjusted significance thresholds were calculated based on the number of comparisons performed, but results were reported using the conventional *p* < 0.05 notation when significance was achieved.

## 3. Results

### 3.1. Effect of BCAAs on Cancer Sarcopenia

**Figure 1:**
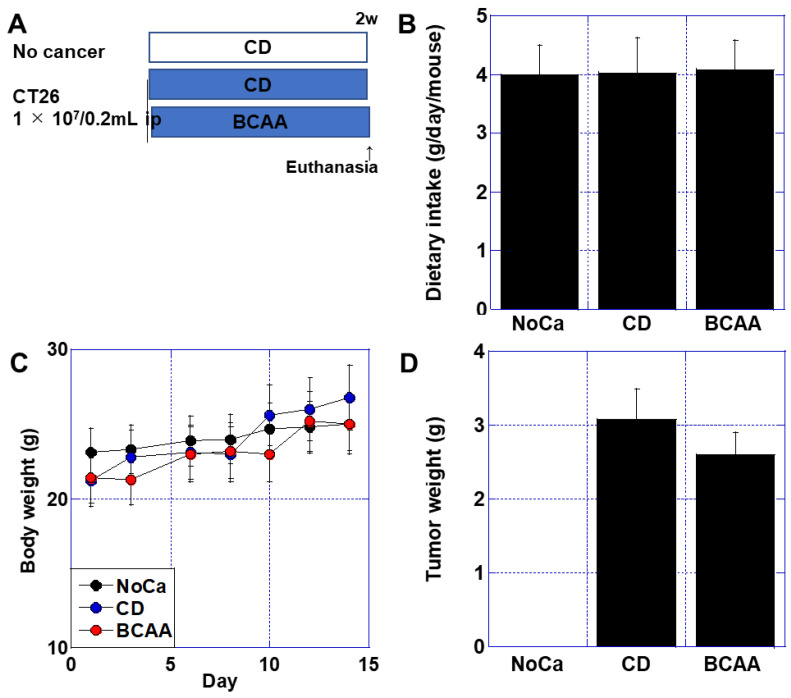
Effect of BCAAs on cancer cachexia. (**A**) An experimental protocol. Mice were divided into 3 groups; NoCa group (fed with CE-2 standard diet, no tumor), CD group (CE-2 diet, CT26 cells ip), and BCAA group (BCAA diet [Table 2], CT26 cells ip). Each group comprised 5 mice. (**B**) Dietary intake. (**C**) Body weight. (**D**) Tumor weight. Error bar, standard deviation from 5 mice. Statistical differences were calculated by ordinary ANOVA with Bonferroni’s correction. ANOVA, analysis of variance; BCAA, branched-chain amino acid; CD, control diet; No Ca, no cancer; ip, intraperitoneal inoculation.

To investigate the effects of BCAAs on cancer-associated cachexia, we first examined its impact using a cachexia model, as BCAAs are known to promote muscle growth in healthy individuals. The mouse cachexia model was established by intraperitoneal inoculation of syngeneic BALB/c mice with CT26 murine colon cancer cells [27]. The control group (Cont) consisted of non-tumor-inoculated mice fed a standard diet (CD), while the tumor-inoculated group was divided into two subgroups: one receiving the CD diet and the other receiving a BCAA-supplemented diet (Figure 1A). No significant differences were observed among the groups in terms of food intake, body weight changes, or tumor weight (Figure 1B–D).

**Figure 2:**
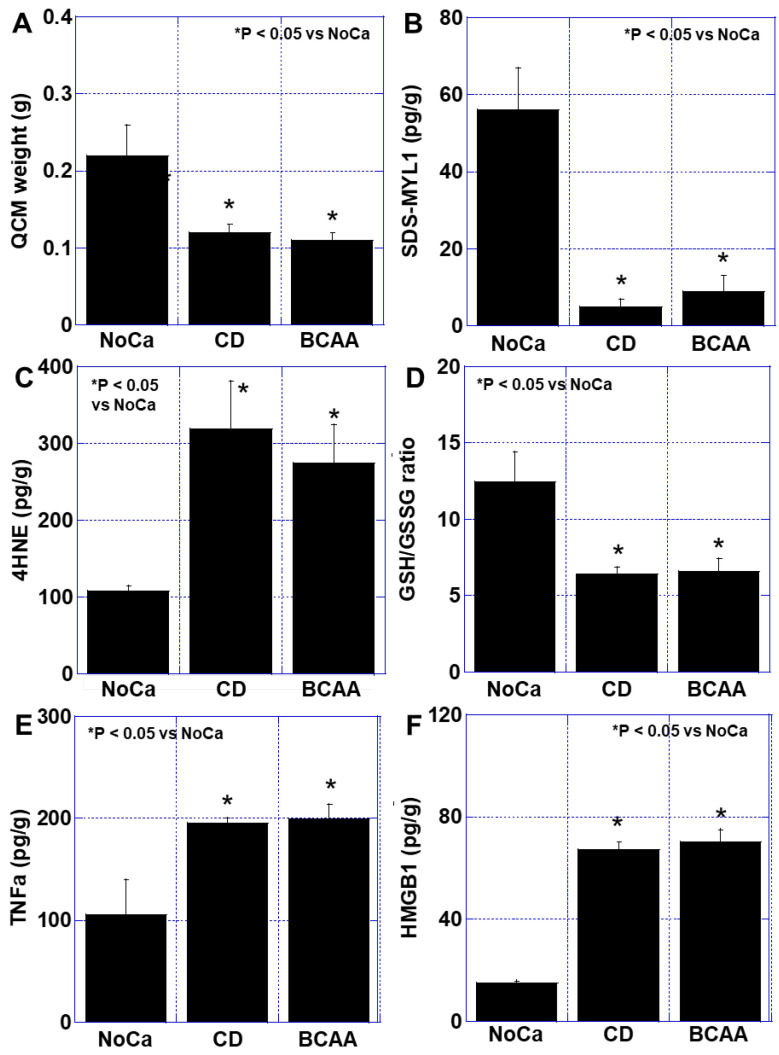
Effect of BCAAs on cancer sarcopenia. (**A**) QCM weight. (**B**) Muscle maturity by SDS-MYL1. (**C**) Muscle oxidative stress by 4HNE. (**D**) Muscle redox by GSH/GSSG ratio. (**E**,**F**) Muscle cytokines; TNFα (**E**) and HMGB1 (**F**). Error bar, standard deviation from 5 mice. Statistical differences were calculated by ordinary ANOVA with Bonferroni’s correction. 4HNE, 4-hydroxynonenal; ANOVA, analysis of variance; BCAA, branched-chain amino acid; CD, control diet; GSH, glutathione; GSSG, glutathione disulfide; HMGB1, high-mobility group box-1; NoCa, no cancer; QCM, quadriceps muscle; SDS-MYL1, SDS-soluble myosin light chain-1; TNF, tumor necrosis factor.

The weight of the quadriceps femoris muscle (QCM) decreased by 48% in the BCAA group compared to the Cont group, with no significant difference observed between the CD and BCAA groups (Figure 2A). Additionally, the sodium dodecyl sulfate-soluble myosin light chain-1 (SDS-MYL1) content, a marker of muscle maturity, was significantly reduced in both the CD and BCAA groups (6 pg/g and 9 pg/g, respectively) compared to the Cont group (56 pg/g) (Figure 2B). Oxidative stress, indicated by elevated levels of 4-hydroxynonenal (4HNE), was significantly increased, while the glutathione/glutathione disulfide (GSH/GSSG) ratio was markedly reduced in both the CD and BCAA groups compared to in the Cont group (Figure 2C,D). Furthermore, the intramuscular concentrations of TNF-α and HMGB1, which are associated with cachexia [13], were significantly elevated in both the CD and BCAA groups compared to in the Cont group, with no significant differences between the CD and BCAA groups (Figure 2E). These findings indicate that BCAA supplementation alone did not mitigate muscle loss or inflammatory responses in the cachexia model.

### 3.2. Effects of BCAAs on Cancer-Related Impairment of Energy Metabolism

**Figure 3:**
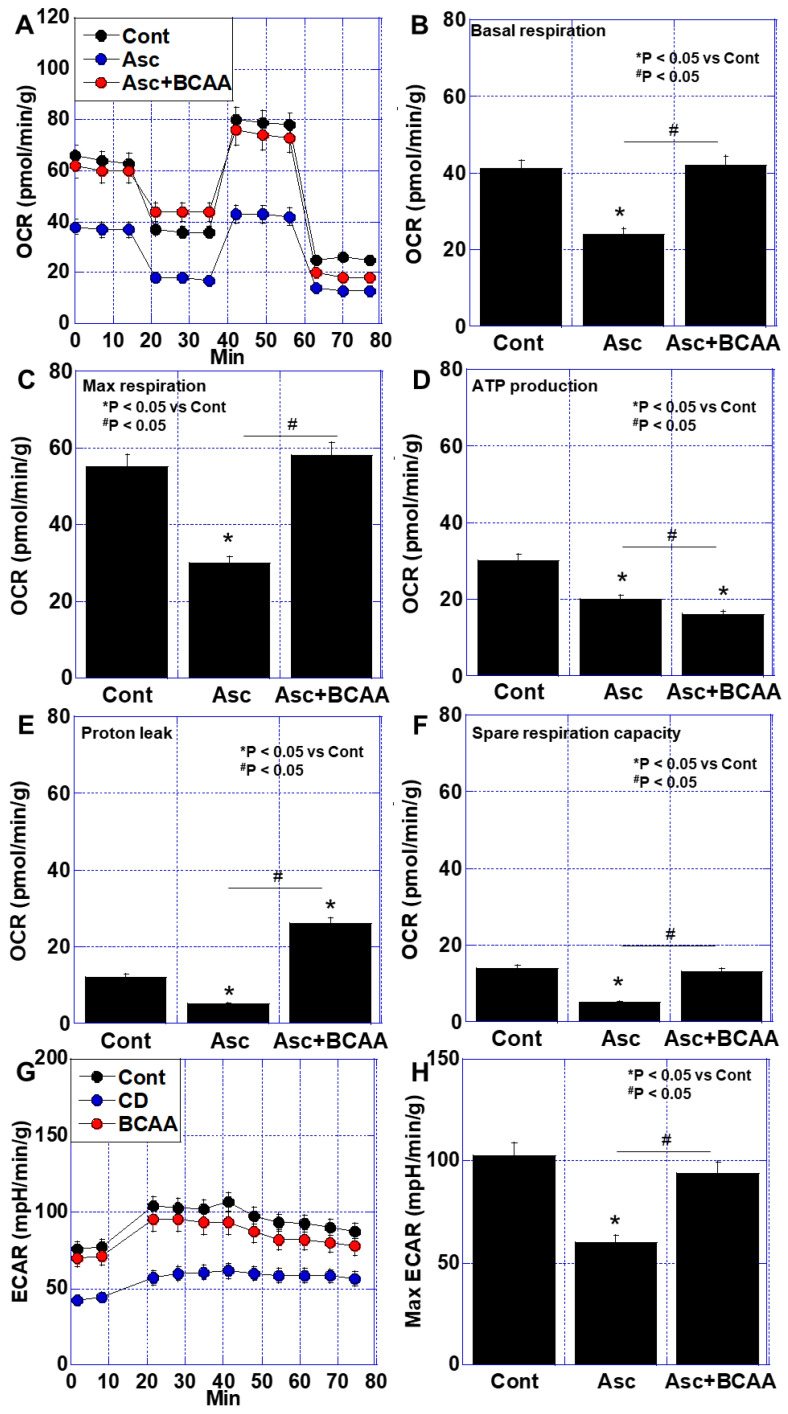
Effect of BCAAs on energy metabolism in an in vitro cachexia model. C2C12 cells (1 × 10^4^) cells were pretreated with cancer ascites (20% *v*/*v*) to fresh DMEM medium. Then, cells were cultured in the mitochondrial stress test medium for 6 h. (**A**) Oxidative phosphorylation by flux analysis. (**B**) Basal respiration. (**C**) Maximum respiration. (**D**) ATP production. (**E**) Proton leak. (**F**) Spare respiration capacity. (**G**) ECAR. (**H**) Maximum ECAR. Error bar, standard deviation from 3 independent trials. Statistical differences were calculated by ordinary ANOVA with Bonferroni’s correction. ANOVA, analysis of variance; Asc, cancer ascites; ATP, adenosine triphosphate; BCAA, branched-chain amino acid; C, control; DMEM, Dulbecco’s Modified Eagle’s Medium; ECAR, Glycolysis by extracellular acidity rate; OCR, oxygen consumption rates.

Next, we investigated the effects of BCAAs on mitochondrial energy metabolism using an in vitro cachexia model [26], in which C2C12 mouse myoblasts were treated with ascites fluid derived from the aforementioned cachexia model (Figure 3). The ascites treatment significantly reduced the basal respiration, maximal respiration, ATP production, and spare respiratory capacity to approximately half the levels observed in the non-ascites control (Cont) group (Figure 3A–F). In the Asc + BCAA treatment group, the basal and maximal respiration recovered to levels comparable to the control. However, ATP production remained lower than that observed with the ascites treatment alone, likely due to an increase in proton leakage (Figure 3E). Additionally, an extracellular acidification rate (ECAR) analysis was performed to evaluate glycolysis and lactate fermentation (Figure 3G,H). The ascites treatment led to a decrease in the ECAR, which was subsequently improved by the BCAA treatment. These results suggest that while BCAA supplementation enhances mitochondrial respiration, its effects on ATP production remain uncertain due to the increased proton leakage.

### 3.3. BCAA Metabolism in the Cachexia Models

**Figure 4:**
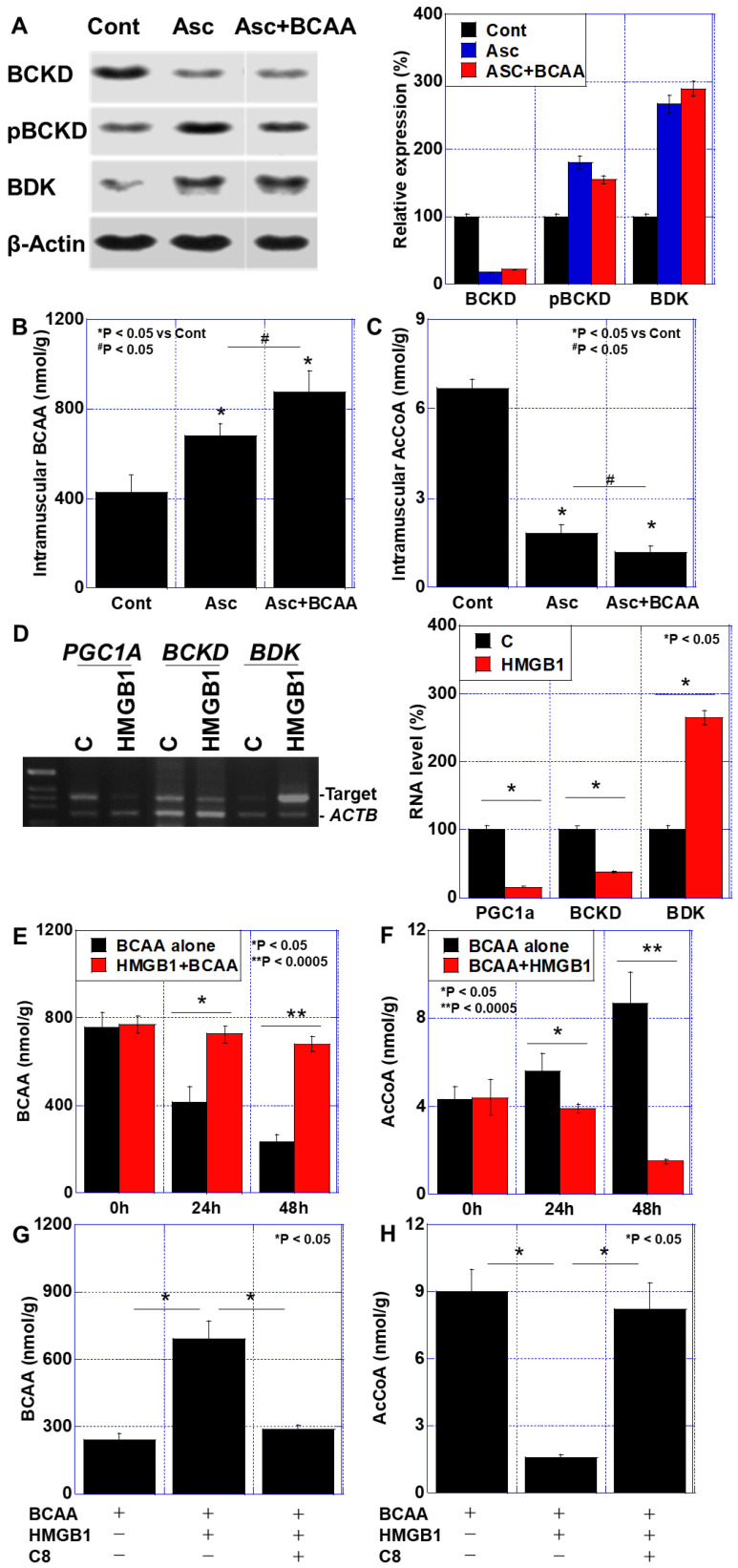
Impaired BCAA metabolism in an in vitro cachexia model. (**A**–**C**) C2C12 cells (1 × 10^6^) were treated with cancer ascites (20% *v*/*v* to fresh DMEM medium) with or without BCAAs (Leu, Ile, and Val, 200 μM each) for 48 h. (**A**) Levels of BCAA metabolism-associated proteins. Right panel: semi-quantification of Western blotting. (**B**) Intramuscular BCAA concentration. (**C**) Intramuscular AcCoA concentration. (**D**–**H**) C2C12 cells were treated with HMGB1 (40 μg/mL) with or without BCAAs (Leu, Ile, and Val, 200 μM each) or C8 (50 μg/mL) for 48 h. (**D**) Expression of BCKD-related genes. Right panel: semi-quantification of RT-PCR. (**E**,**F**) Effect of HMGB1 on intramuscular BCAA (**E**) and AcCoA (**F**) concentrations. (**G**,**H**) Effect of C8 on intramuscular BCAA (**G**) and AcCoA (**H**) concentrations. Error bar: standard deviation from 3 independent trials. Statistical differences were calculated by ordinary ANOVA with Bonferroni’s correction. AcCoA, acetyl coenzyme A; ACTB, β-actine; ANOVA, analysis of variance; Asc, cancer ascites; BCAA, branched-chain amino acid; BDK, branched-chain ketoacid dehydrogenase kinase; BCKD, branched-chain α-ketoacid dehydrogenase; C8, caprylic acid; Cont, control; C, control; DMEM, Dulbecco’s Modified Eagle’s Medium; HMGB1, high-mobility group box-1; pBCKD, phosphorylated; PGC1A, peroxisome proliferator-activated receptor-γ coactivator-1α; RT-PCR, reverse transcription—polymerase chain reaction.

Given the previously reported metabolic impairments of BCAAs in diabetes mellitus and cancer [29,30], we examined BCAA metabolism in the in vitro cachexia model (Figure 4). Specifically, we analyzed alterations in the branched-chain α-ketoacid dehydrogenase (BCKD) complex, which is involved in BCAA catabolism, and its regulatory enzyme, branched-chain α-ketoacid dehydrogenase kinase (BDK) (Figure 4A). The ascites treatment led to a decrease in the BCKD protein levels, an increase in phosphorylated (inactive) BCKD, and the upregulation of BDK expression. The BCAA treatment did not restore these alterations. Moreover, the intramuscular BCAA levels were significantly elevated following the ascites treatment and were further increased in the Asc + BCAA treatment group (Figure 4B). In contrast, the intramuscular acetyl AcCoA levels were reduced following the ascites treatment, and this reduction was exacerbated by the combined Asc + BCAA treatment (Figure 4C). These findings suggest that impaired BCAA metabolism in cancer cachexia leads to its accumulation and limits its protective effects on skeletal muscle.

To further explore the underlying mechanisms, we examined the role of inflammatory cytokines in inhibiting BCAA utilization. The treatment of the C2C12 myoblasts with HMGB1 (40 μg/mL) led to the downregulation of *PGC1α* and *BCKD* gene expression, while *BDK* was upregulated (Figure 4D). HMGB1 also induced BCAA retention within the C2C12 cells (Figure 4E) while significantly reducing the AcCoA levels (Figure 4F). These findings indicate that HMGB1 suppresses BCAA metabolism by inhibiting BCKD activity.

Next, we investigated whether inhibiting BDK could restore BCAA metabolism. C8, a known BDK inhibitor [31], was used as a treatment. The co-treatment of the C2C12 cells with HMGB1 (40 μg/mL) and C8 (50 μg/mL) reversed HMGB1-induced BCAA retention (Figure 4G) and restored the AcCoA levels (Figure 4H). These results suggest that targeting BDK with C8 may enhance BCAA metabolism and improve muscle preservation in cancer cachexia.

### 3.4. Effects of C8 on HMGB1-Induced Skeletal Muscle Impairment

**Figure 5:**
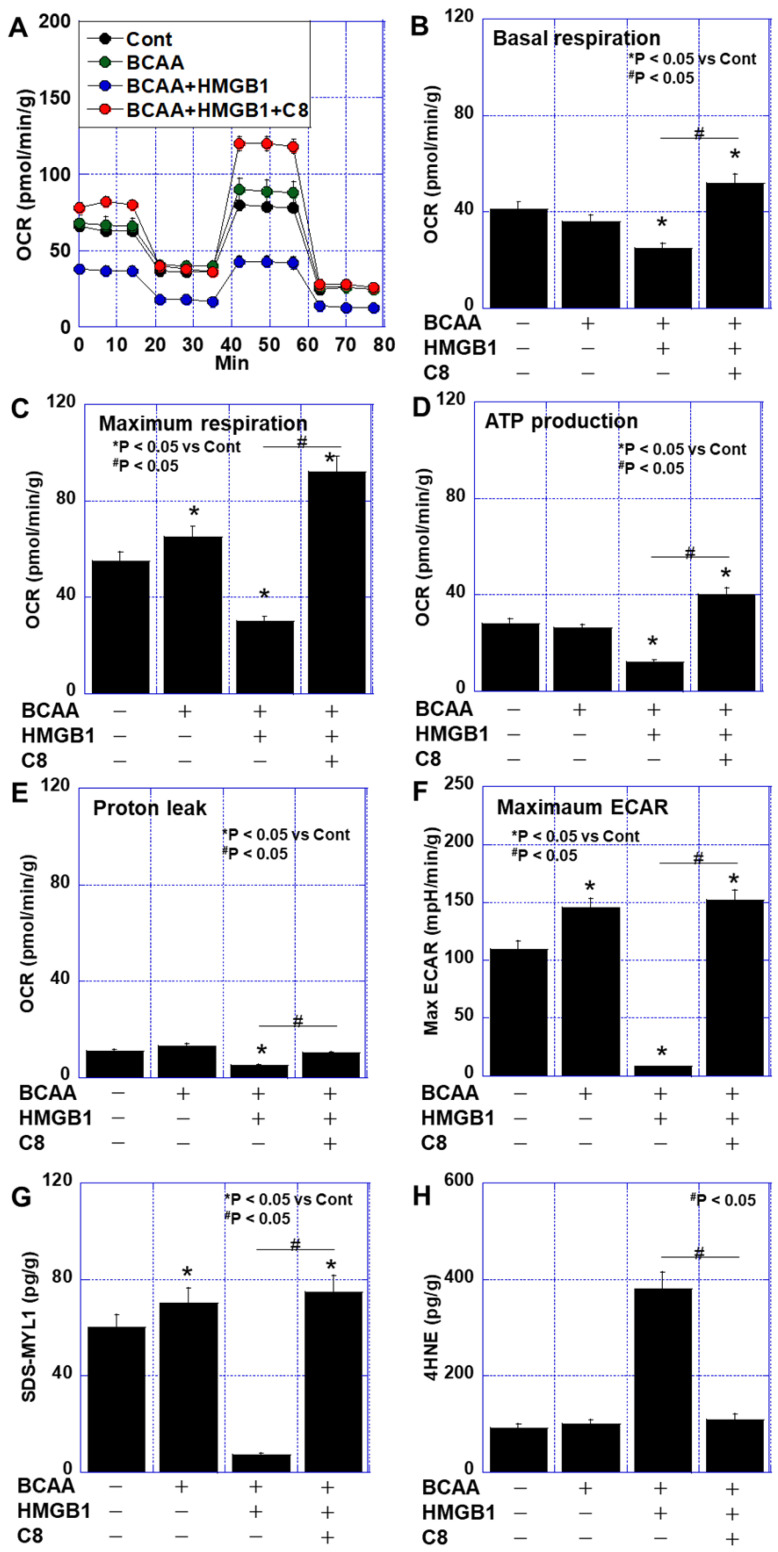
Effect of BCAAs combined with HMGB1 and/or C8 on energy metabolism in C2C12 cells. C2C12 cells were treated with BCAAs (Leu, Ile, and Val, 200 μM each), HMGB1 (40 μg/mL), and/or C8 (50 μg/mL) for 48 h. For flux assay, cells were then cultured in the mitochondrial stress test medium for 6 h. (**A**) Oxidative phosphorylation by flux analysis. (**B**) Basal respiration. (**C**) Maximum respiration. (**D**) ATP production. (**E**) Proton leak. (**F**) Maximum ECAR. (**G**) SDS-MYL1. (**H**) 4HNE. Error bar: standard deviation from 3 independent trials. Statistical differences were calculated by ordinary ANOVA with Bonferroni’s correction. 4HNE, 4-hydroxynonenal; ANOVA, analysis of variance; ATP, adenosine triphosphate; BCAA, branched-chain amino acid; C, control; C8, caprylic acid; ECAR, extracellular acidity rate; HMGB; HMGB1, high-mobility group box-1; OCR, oxygen consumption rates; SDS-MYL1, SDS-soluble myosin light chain-1.

To further evaluate the effects of C8 on skeletal muscle function, we cultured C2C12 myoblasts in normal medium and examined the impact of BCAAs on energy metabolism (Figure 5A–F). The HMGB1 treatment suppressed basal respiration and ATP production, but co-treatment with C8 restored these functions to levels exceeding those of the control group (Figure 5B–D). Maximum respiration, which was enhanced by BCAAs alone but suppressed by HMGB1, was also restored by C8 (Figure 5C). Proton leakage, which was reduced in the BCAA + HMGB1 group, returned to control levels following the C8 treatment (Figure 5E). Additionally, the C8 treatment restored the SDS-MYL1 levels and reduced oxidative stress (4HNE levels) in the HMGB1-treated cells (Figure 5G,H). These findings suggest that C8 alleviates HMGB1-induced impairments in skeletal muscle metabolism and function, thereby enhancing the muscle-protective effects of BCAAs in cancer cachexia.

### 3.5. Effect of BCAAs When Combined with 5FU Treatment in a Mouse Cachexia Model

**Figure 6:**
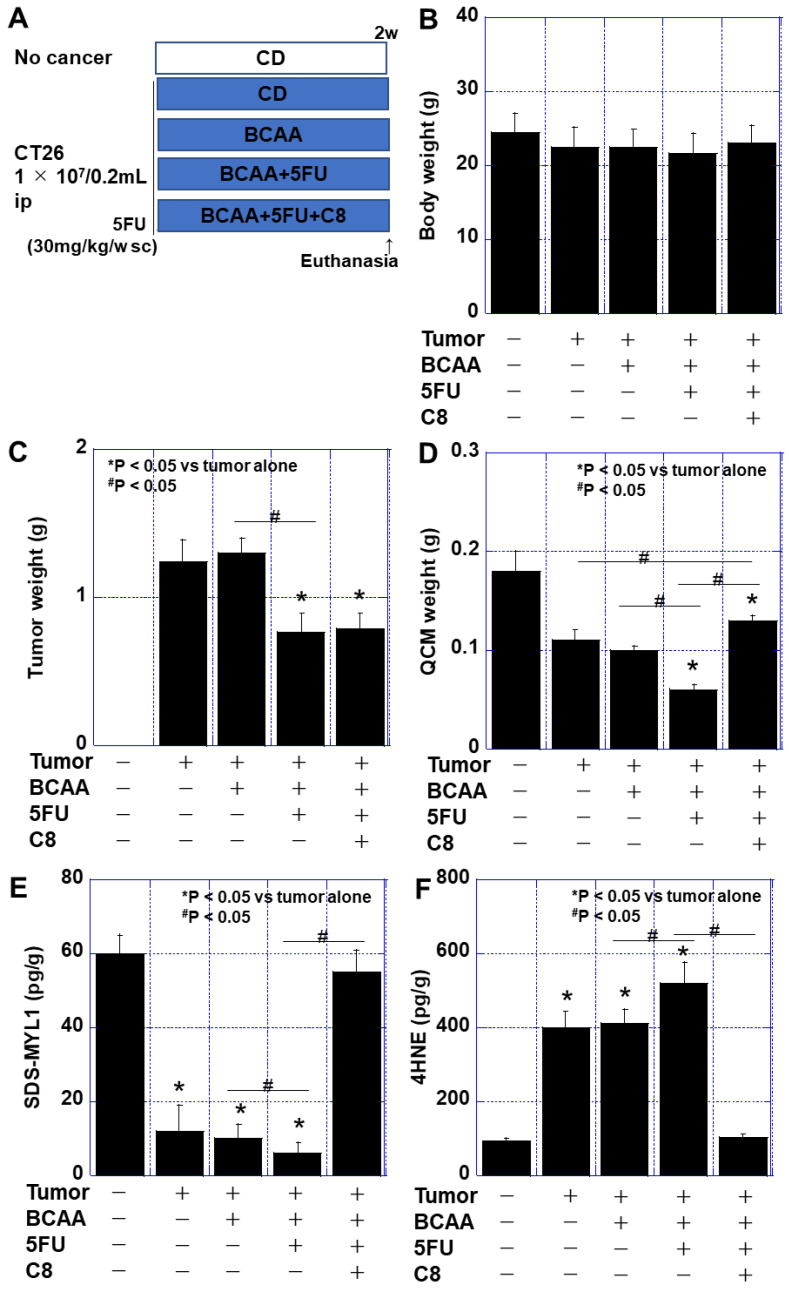
Effect of BCAAs on cancer sarcopenia in 5FU-administered mice. (**A**) An experimental protocol. Mice were divided into 5 groups: no cancer group (fed CE-2 standard diet, no tumor), CD group (fed BCAA diet [Table 2], CT26 cells ip), BCAA group (fed BCAA diet [Table 2], CT26 cells ip), BCAA + 5FU group (fed BCAA diet [Table 2], 5FU [30 mg/kg/w sc], CT26 cells ip) and BCAA + 5FU + C8 group (fed BCAA + C8 diet [Table 2], 5FU [30 mg/kg/w sc], CT26 cells ip). Each group comprised 5 mice. (**B**) Body weight. (**C**) Tumor weight. (**D**) QCM weight. (**E**) Muscle maturity by SDS-MYL1. (**F**) Muscle oxidative stress by 4HNE. Error bar: ordinary deviation from 5 mice. Statistical differences were calculated by standard ANOVA with Bonferroni’s correction. 4HNE, 4-hydroxynonenal; 5FU, 5-fluorouracil; ANOVA, analysis of variance; BCAA, branched-chain amino acid; C8, caprylic acid; CD, control diet (CE-2); QCM, quadriceps muscle; SDS-MYL1, SDS-soluble myosin light chain-1.

Finally, we investigated the impact of chemotherapy on sarcopenia and the potential protective effects of BCAA supplementation in this context (Figure 6). 5FU (30 mg/kg/week) was administered to mice with cachexia receiving a BCAA-supplemented diet. No significant differences were observed in body weight among the five experimental groups (Figure 6B). However, the tumor weight was reduced by 55% in the BCAA + 5FU group compared to that in the tumor-alone group (Figure 6C). The quadriceps femoris muscle weight and SDS-MYL1 levels were not rescued by BCAA supplementation alone and were further reduced by 5FU. However, the addition of C8 effectively restored muscle maturity and reduced oxidative stress (Figure 6E,F). These findings suggest that while BCAAs alone do not protect against chemotherapy-induced sarcopenia, their combination with C8 enhances muscle preservation.

## 4. Discussion

In this study, we examined the effects of BCAAs on cancer-associated sarcopenia and found that BCAAs did not exert the expected protective effects on muscle that are typically observed in healthy individuals due to the impairment of BCAA metabolism via BCKD inactivation. An inflammatory cytokine, HMGB1, inactivated BCKD, which was restored by C8 treatment.

BCAAs are widely recognized as effective supplements for enhancing muscle mass and function, particularly when combined with exercise [32,33,34]. Among BCAAs, Leu has garnered particular attention for its ability to stimulate acute anabolic responses [35]. Additionally, low BCAA concentrations in individuals with sarcopenia suggest a potential therapeutic role for BCAA supplementation in muscle preservation [36]. Furthermore, BCAAs have been shown to upregulate gene expression related to mitochondrial biogenesis and function in skeletal muscle affected by cancer sarcopenia [37]. However, the effects of BCAA supplementation in conditions such as sepsis, cancer, diabetes, and obesity remain uncertain, necessitating further investigation [38].

This study demonstrated that impaired BCAA utilization in cancer patients compromises the muscle-protective effects of BCAAs. Elevated blood BCAA levels have been observed in cancer patients, suggesting a disruption in BCAA metabolism [39]. Although the exact mechanisms remain unclear, several factors have been implicated. Thyroid hormone, which is elevated during starvation, has been shown to promote the expression of BDK, leading to impaired BCAA utilization [40]. Notably, increased blood thyroid hormone levels have been reported in cancer patients [41,42]. Moreover, BDK has been identified as a key regulator of metabolic reprogramming in cancer cachexia [30]. The suppression of BCKD activity may contribute to the observed impairment in BCAA utilization in cancer patients.

HMGB1 functions as an autocrine/paracrine growth factor in various cancers, promoting malignant phenotypes and disease recurrence [43]. Additionally, HMGB1 has been shown to exacerbate cancer-associated sarcopenia [13,14,17]. In this study, BCAA supplementation did not suppress the expression of either TNFα or HMGB1. Notably, BCAAs have been reported to promote cytokine production when accumulated in excess [29], which is consistent with our findings. Furthermore, we demonstrated that HMGB1 promotes the expression of BDK by downregulating PGC1α, thereby inhibiting BCKD and reducing BCAA metabolism.

BCAA metabolism consists of two key processes: the conversion of BCAAs to branched-chain α-keto acids (BCKAs) via cytoplasmic or mitochondrial branched-chain aminotransferase (BCAT), followed by BCKA degradation in mitochondria by BCKD. This degradation produces acetyl-CoA, propionyl-CoA, and 2-methylbutyryl-CoA, which are subsequently utilized for ATP generation [44]. In this study, treatment with cancer ascites led to decreased BCKD expression, increased BDK expression, and enhanced phosphorylation of BCKD, resulting in impaired BCAA metabolism. A similar inactivation of the BCKD complex has been observed in multiple organs (liver, muscle, and kidneys) in diabetes, leading to organ damage [29]. Dysfunctional BCKD activity has also been linked to cardiac dysfunction, systolic ventricular dilation, and abnormal gene expression in the myocardium [45]. Impaired BCAA catabolism, characterized by BCAA and BCKA retention due to decreased BCKD function, results in mitochondrial oxidative stress, redox imbalance, inflammation, tissue fibrosis, and increased cytokine production via macrophage hyperactivation [29]. Our findings indicate that such BCAA catabolic impairment hinders the beneficial effects of BCAA supplementation in ameliorating cancer sarcopenia.

BCKD dysfunction can arise not only from genetic disorders such as maple syrup urine disease but also from factors including fatty acids, inflammatory cytokines, oxidative stress, and elevated insulin levels [44,46]. Our data suggest that HMGB1 in cancer ascites suppresses PGC1α expression while activating BDK, thereby impairing BCAA utilization in C2C12 cells. Extracellular HMGB1 has previously been reported to downregulate PGC1α expression and inhibit mitochondrial biogenesis [47,48]. In contrast, cytoplasmic HMGB1 has been shown to bind to cytoplasmic toll-like receptor-9 (TLR9) to promote PGC1α expression and mitochondrial biogenesis [49]. As an inflammatory cytokine, HMGB1 is believed to primarily exert the former effect.

Although the relationship between inflammatory cytokines and impaired BCAA metabolism has not been fully elucidated, our findings suggest that this mechanism may contribute to the pathogenesis of cancer sarcopenia. HMGB1 has been implicated in cancer-associated sarcopenia [13,27,50,51,52,53], cancer-related cardiac dysfunction [26,52], and skeletal muscle aging [17]. The induction of BCAA metabolic impairment by HMGB1 may be a contributing factor to these disorders. HMGB1 has a mutual induction relationship with inflammatory cytokines such as TNFα, forming a vicious cycle. Future investigations into whether cytokines other than HMGB1 are involved in BCAA metabolic disorders will be useful in elucidating the mechanism of cancer sarcopenia.

To maximize the therapeutic effects of BCAAs in cancer-associated sarcopenia, it is essential to address the underlying impairment in BCAA catabolism. The inhibition of BDK, the enzyme responsible for phosphorylating and inactivating BCKD, or the overexpression of protein phosphatase-1K (PPM1K), which dephosphorylates and activates BCKD, has been reported to reduce circulating BCAA levels, alleviate hepatic steatosis, and improve glucose tolerance [53]. To find food components that restore BCKD activity, we investigated the effects of food ingredients that we have previously investigated (lauric acid, capric acid, caprylic acid, berberine, and pterostilbene) and found that C8 restored BCKD activity. We also found that C8 has been reported to possess BDK inhibitory activity [31]. Our previous studies have demonstrated that C8 exerts a strong protective effect on skeletal muscle [28]. Additionally, C8 enhances mitochondrial turnover and improves mitochondrial quality control. The findings of this study suggest that C8 restores BCKD activity by inhibiting BDK, thereby ameliorating impaired BCAA utilization. These results indicate that the muscle-protective effects of BCAAs in cancer patients may be enhanced by molecularly targeting BDK.

Previous studies have reported that glucocorticoids and PPARα ligands suppress BDK expression [40,54]. Investigating their effects on cancer sarcopenia, as well as developing more potent BDK inhibitors, could provide more effective therapeutic strategies for managing cancer sarcopenia. Although targeting different molecules, the inhibition of mitochondrial pyruvate carrier has been shown to improve BCAA metabolism in diabetic patients [55].

Given the importance of BDK inhibition in restoring BCAA metabolism, we explored additional mechanisms that may further optimize BCAA utilization. Notably, BCKD activity is suppressed by reactive nitrogen species produced by cytokines [56]. While cystine and theanine have been reported to independently mitigate sarcopenia by reducing oxidative stress [19], they may also attenuate BCKD inhibition, thereby improving BCAA utilization. Unlike C8, cystine and theanine likely enhance BCAA metabolism through distinct mechanisms, potentially amplifying their therapeutic effects against sarcopenia.

In the mouse cachexia model, treatment with 5FU reduced the tumor weight but simultaneously increased oxidative stress in skeletal muscle, leading to suppressed QCM weight and reduced muscle maturity. These findings suggest that anticancer drug treatment exacerbates sarcopenia through oxidative stress induction. However, in C2C12 myoblasts treated with cancer ascites, BCAA supplementation promoted oxidative phosphorylation and glycolysis but failed to increase ATP production due to excessive proton leakage, ultimately resulting in an insufficient protective effect on muscle weight and maturity. In contrast, C8 supplementation effectively ameliorated 5FU-induced sarcopenia when combined with BCAAs. These results suggest that BCAA supplementation, when coupled with metabolic enhancement, may also be beneficial in mitigating chemotherapy-induced sarcopenia.

However, chemotherapy-induced sarcopenia remains an underexplored area, and this study only examined the effects of 5FU. Future investigations should assess whether other chemotherapeutic agents exert similar effects and whether metabolic enhancement strategies remain effective across a broader range of anticancer treatments. Additionally, given that this study was conducted using a murine model, further research is needed to confirm whether similar metabolic alterations occur in human cancer patients.

We used an in vitro cachexia model to investigate impaired BCAA utilization. Many studies have reported that humoral factors such as inflammatory cytokines play an important role in the development of cachexia [7,8,9]. We have obtained similar results [13,14]. Meanwhile, the cancer ascites generated in our mouse cachexia model contains various inflammatory cytokines, which correlate with the development of cachexia [26,27]. To establish an in vitro cachexia model, we devised a method to treat C2C12 cells with cachexia-related humoral factors in ascites by mixing the cancer ascites with the culture medium [26]. This system has, so far, produced results that correlate well with the mouse model, and is considered to be useful as an in vitro model of sarcopenia due to cachexia [19,26,52]. One problem is that it is not yet fully clear what factors are contained in the ascites other than the inflammatory cytokines already detected. It has also not been determined to what extent the components in the blood of tumor-bearing bodies and the components in the cancer ascites are the same. This should be elucidated in the future using metabolomics or proteomics.

At the systemic level, hepatocytes play a crucial role in BCAA metabolism. In this study, we did not examine BDK expression changes in hepatocytes, highlighting an area for further investigation. Additionally, we did not monitor alterations in BCKA concentrations. Since excessive BCKA accumulation has been associated with potential toxicity and organ damage [57], future studies should incorporate high-performance liquid chromatography (HPLC) analysis to assess the BCKA levels more precisely. Furthermore, early rodent studies suggested strong muscle-protective effects of BCAAs; however, recent detailed investigations have yielded conflicting results [30]. Considering this discrepancy, the findings of our study, which are based on a murine model, require further validation in human subjects.

## 5. Conclusions

Our findings suggest that the therapeutic effects of BCAAs in cancer-associated sarcopenia could be enhanced to levels comparable to those observed in healthy individuals by normalizing BCAA metabolism. Addressing BCAA metabolic impairments may expand dietary intervention options for cancer sarcopenia, offering a novel strategy for improving patient outcomes.

## Figures and Tables

**Table 1:** Components of media for treatment.

Component	Medium		
	D-MEM	Ascites Added ^1)^	CM Added ^2)^
Glucose (mg/dL)	450 ± 2	361 ± 8	378 ± 6
Pyruvate (mg/dL)	11 ± 0.1	9 ± 1	9 ± 1
Glutamine (mg/dL)	58 ± 0.2	49 ± 4	50 ± 3
Lactate (pmol)	0	7.4 ± 1.4	1.2 ± 0.2
HMGB1 (μg/mL)	ND	18 ± 0.9	ND
TNFα (pg/mL)	ND	12 ± 0.2	ND

^1)^ Ascites was collected from mice 2 weeks after inoculation of CT26 mouse colon cancer cells (1 × 10^7^) intraperitoneally. Ascites was added at 20% *v*/*v* to fresh D-MEM culture medium supplemented with 10% FBS. ^2)^ Cultured medium (CM) of CT26 cells (1 × 10^8^) after 48 h was added at 20% *v*/*v* to fresh D-MEM culture medium supplemented with 10% FBS. D-MEM, Dulbecco’s Modified Eagle’s Medium; HMGB1, high-mobility group box-1; TNFα, tumor necrosis factor-α; ND, not detected.

**Table 2:** Food ingredients.

Ingredient	Control Diet	BCAA Diet	BCAA + C8 Diet
Moisture (%)	8.83	8.57	8.66
Crude protein (%)	25.13	24.38	24.65
Crude fat (%)	4.92	4.77	4.65
Crude fiber (%)	4.42	4.28	4.21
Crude ash (%)	6.86	6.65	6.54
NFE (%)	49.84	48.34	47.35
Valine (%)	-	1	1
Laucine (%)	-	1	1
Isoleucine (%)	-	1	1
Energy (kcal)	334.2	345.874	371.85

C8, caprylic acid; BCAA, branched-chain amino acid.

**Table 3:** Primary sets, antibodies, and enzyme-linked immunosorbent assay (ELISA) kits.

Gene	Accession No.	Upper Primer	Lower Primer
*PGC1A*	BC156323.1	aaggatgcgctctcgttcaa	ttcgtttgacctgcgcaaag
*BDK*	CR542093.1	ctcggtacctgcagcaagaa	tggcatagggatgaagggga
*ACTB*	NM_007393.5	acaatgagctgcgtgtggcc	agggacagcacagcctggat
**Target**	**Cat. No.**	**Company**	**Adderess**
Antibodies			
BCKD	ab126173	Abcam	Cambridge, MA, USA
phospho-BCKD, pS293	ab200577	Abcam	Cambridge, MA, USA
BDK	ab128935	Abcam	Cambridge, MA, USA
β-Actin	sc-47778	Santa-Cruz	Dallas, TX, USA
ELISA kit			
MYL1	CSB-EL015305MO	Cusabio Biotech	Houston, TX, USA
HMGB1	326078738	Shino Test	Sagamihara, Japan
Mouse TNF-α	MTA00B	R&D Systems	Minneapolis, MN, USA
GSH/GSSG	CB-P050-K	Creative Biolabs	Shirley, NY, USA
4HNE	STA-838	Cell Biolabs	San Diego, CA, USA
BCAA	MET-5056	Cell Biolabs	San Diego, CA, USA
AcCoA	RE10014	Reed Biotech	Wuhan, China

4HNE, 4-hydroxynonenal; AcCoA, acetyl coenzyme A; BCAA, branched-chain amino acid; BCKD, branched-chain alpha-keto acid dehydrogenase α subunit; BDK, BCKD kinase; GSH, glutathione; GSSG, glutathione disulfide; HMGB1, high-mobility group box-1; MYL1, myosin light chain-1; TNF, tumor necrosis factor.

## Data Availability

The data are contained within the article.

## Abbreviations

BCAA	branched-chain amino acid
BCKD	branched-chain α-ketoacid dehydrogenase
HMGB1	high-mobility group box-1
QCM	quadriceps femoris muscle
SDS-MYL1	sodium dodecyl sulfate-soluble myosin light chain-1
4HNE	4-hydroxynonenal
TNF	tumor necrosis factor
BDK	BCKD kinase
AcCoA	acetyl coenzyme A
PGC1α	peroxisome proliferator-activated receptor-γ coactivator-1α
C8	caprylic acid
